# Evaluation of the diagnostic accuracy of the ReLASV Pan-Lassa Antigen Rapid Test for Lassa Fever in Nigeria

**DOI:** 10.1371/journal.pgph.0004405

**Published:** 2025-04-21

**Authors:** Hanesh F. Chi, Johnson Etafo, Fritz Fonkeng, Gbenga-Ayeni Olufunke, Ronke Ireneh, Chukwuyem Abejegah, Sampson Owhin, Daniel G. Bausch, Aurelia Vessiere, Emmanuel Agogo, Devy M. Emperador, Adedosu A. Nelson

**Affiliations:** 1 FIND, Geneva, Switzerland; 2 Federal Medical Centre Owo, Owo, Nigeria; 3 London School of Hygiene and Tropical Medicine, London, United Kingdom; University of Ottawa Faculty of Medicine, CANADA

## Abstract

Lassa fever is a zoonotic disease found in several countries across West Africa, with estimates of up to 300,000 infections and 10,000 deaths yearly. The highest incidence is in Nigeria. Suspected cases are often seen in areas with limited infrastructure and diagnostics capacity, hence the availability of an accurate rapid diagnostic test (RDT) that could be used in the community would be an important public health tool. Unfortunately, few RDTs for Lassa fever exist and have not been thoroughly validated. Toward that end, we conducted a Phase 2 performance evaluation to assess the diagnostic accuracy of the ReLASV Pan-Lassa Antigen Rapid Test (Zalgen Labs, Frederick, MD, USA) using archived, frozen whole blood, plasma, and serum samples collected from individuals in Nigeria to determine its suitability for widespread use as a screening tool for Lassa fever. The overall performance of this RDT was measured against the reference test, the Altona RealStar LASV real-time reverse transcription polymerase chain reaction 2.0 (Altona Diagnostics, Hamburg, Germany). The sensitivity and specificity of the ReLASV Pan-Lassa Antigen Test were 65% and 50.7%, respectively. The low diagnostic accuracy indicated in our and other independent evaluations of the ReLASV Pan-Lassa Antigen Rapid Test suggests that this test, at least until further developed, refined, and validated, is not suitable for making critical diagnostic or treatment decisions for Lassa fever, at least for lineages that commonly circulate in Nigeria. These findings underscore the importance of thoroughly assessing the performance characteristics of tests to ensure their reliability and accuracy.

## Introduction

Lassa virus (LASV) is a zoonotic high-consequence priority pathogen under the World Health Organization (WHO) R&D Blueprint [1]. The virus is estimated to infect up to 300,000 persons annually across West Africa, causing a potentially severe viral hemorrhagic disease known as Lassa fever (LF) [2]. Ondo State remains a major epicenter of Lassa fever in Nigeria, consistently reporting high case numbers and mortality rates. Since 2020, the state has recorded about 9,400 suspected cases, 1,779 confirmed cases, and 261 deaths. With an estimated case fatality rate of over 14% in the state, Lassa fever remains one of the deadliest infectious diseases in the region. The disease follows a seasonal pattern, with outbreaks peaking during the dry season (November to April) [3,4,5]. Effective patient management and control measures for LF are highly dependent on accurate and timely diagnosis. Because the presentation of the disease is usually non-specific, making it difficult to diagnose on clinical grounds alone, laboratory confirmation is imperative. However, although LF can be diagnosed by a multitude of modalities, including virus cell culture, molecular diagnostics such as the polymerase chain reaction (PCR), and immunoassays to detect viral antigen and/or LASV-specific antibodies, there is a dearth of well-validated commercially available assays. Diagnosis is further complicated by the fact that LF is typically seen in rural settings where laboratory infrastructure and diagnostics capacity, including the presence of trained laboratory personnel, are limited, especially for a high-consequence pathogen requiring special biosafety precautions [6].

Real-time PCR to detect LASV RNA is currently the most widely used laboratory method for diagnosis of LF, and is considered the “gold standard,” but is still not widely accessible. Rapid diagnostic tests (RDTs) to detect LASV antigen are a promising alternative to provide rapid case identification and guide public health response in decentralized settings due to their simplicity, rapidity, and adaptability for point-of-care use [7,8]. However, there are few RDTs for LF on the market, with limited independent performance data on the ones that do exist [9]. Toward that end, we assessed the performance of a LASV antigen RDT to determine its suitability for widespread use as an essential tool for the rapid diagnosis of LF.

## Materials and methods

### Study design and sample collection

This was an observational, retrospective, phase 2 [10] diagnostic accuracy study to determine the performance of one LASV antigen RDT using archived, frozen blood samples collected from individuals in Nigeria from October 2021 to September 2022. The evaluation was conducted from 22^nd^ October 2022 to 25^th^ November 2022 at the Federal Medical Center Owo (FMCO), Infection Control and Research Laboratory in Ondo State, Nigeria. The study samples used were archived frozen LASV-positive and LASV-negative whole blood, plasma and serum samples previously collected from individuals in Nigeria with clinical suspicion of LF under the Integrated Biobank Initiative [11] conducted by the international organisation FIND, Geneva, Switzerland, in collaboration with (FMCO). Diagnosis of LF at FMCO is conducted systematically on these individuals who presented to the outpatient and inpatient facilities at the study site with symptoms of Lassa fever through a combination of historical, clinical, and laboratory assessments following the National Guidelines for Lassa Fever Case Management [12]. All participants provided documented informed consent (18 years or older), assent (12-17 years, accompanied by the consent of a parent/legal guardian), or consent of both parents or the parent who had primary responsibility for children below 12 years, as appropriate, for the use of leftover samples for further research purposes. Individuals who were not competent to provide informed consent, as well as individuals with anatomical or health conditions contraindicating blood or bodily fluid collection, were excluded, as were participants for whom effective follow-up was deemed to be unachievable (e.g., those who lived elsewhere or were planning to relocate). In the event of the death of a patient before diagnosis, enrolment was not possible as these samples were collected as part of a separate study, which involved follow-up of confirmed LF cases. Eligible samples for the study included non-haemolyzed whole blood and plasma collected in tubes containing ethylenediaminetetraacetic acid (EDTA), and serum, all maintained at −20°C or lower within 24 hours of collection. These samples were thawed on ice prior to testing.

### Investigational product

The index test evaluated in this study was the ReLASV Pan-Lassa Antigen Rapid Test (ReLASV Ag-RT, Zalgen Labs, Frederick, MD, USA), a point-of-care, *in vitro* RDT designed to detect the most prevalent LASV lineages (II, III and IV), using a cocktail of polyclonal antibodies raised against LASV recombinant nucleoproteins [8]. The ReLASV Pan-Lassa Antigen Rapid Test is performed as a dipstick immunoassay. A whole blood, plasma, or serum sample is added to the Sample Pad. Inserting the dipstick into a test tube containing the sample buffer initiates the sample flow through the reagent pads and across the nitrocellulose membrane. As the anti-LASV NP antibody Test stripe captures these complexes, the deposition of the gold conjugate generates a pink to dark red signal corresponding to the concentration of LASV NP antigen in the sample. Visual interpretation of test and control bands is made between 15-25 minutes of development time [13].

We measured the performance of this RDT against the reference test, the Altona RealStar LASV qRT-PCR 2.0 (Altona Diagnostics, Hamburg, Germany), an *in vitro* real-time PCR diagnostic test currently used as the gold standard for the qualitative detection of LASV-specific RNA [14], following the manufacturers’ instructions for both tests. The PCR assay targets LASV GPC and L genes, and cycle thresholds are applied according to the manufacturer’s guidelines.

### Testing procedures

Sample panels for index and reference testing were prepared by an independent operator and reference test results and clinical information were blinded to the readers of the index test. Testing was performed by highly skilled, well-trained laboratorians at the Lassa fever laboratory at FMCO. The result for each test performed was verified by a second reader before recording. In case of discrepancy, a third reader was included. Any invalid test runs (e.g., no internal control signal/band) were repeated once within the same freeze-thaw cycle on the same day. If the repeat run remained invalid, the sample was reported as such. Valid index test results that were discrepant to the reference result were not repeated.

### Statistical considerations

The sample size was calculated using the methodology described in [15], based on an expected sensitivity of 85% and specificity of 90% of the index test in comparison to the reference test, as discussed previously [8], and powered to obtain point estimates of pooled sensitivity and specificity with a 95% confidence interval (CI) based on Wilson’s score method, of total width +/- 20% with 80% power. The primary endpoint was the sensitivity and specificity of the index test when compared with the reference test. Point estimates of diagnostic accuracy metrics were calculated for subgroups of sample type, disease severity defined in [12], Ct values of the reference test (categorized as all Ct < 35, all Ct < 30, all Ct < 25), and sex to assess performance of the index test associated with these parameters. The statistical significance of the difference in the distribution of index test results between groups was assessed using a Chi^2^ Test or Fisher’s Exact Test, where appropriate, and was set at 0.05. Statistical analyses were performed using R Statistical Software (version 4.3.1; The R Foundation for Statistical Computing). All the items of the STARD Checklist 2015 (S1 Table) were considered during the development of this manuscript.

### Ethics statement

Ethics approval (Ref: FMC/OW/380/VOL.CXLIX/137, 21 April 2022) was obtained before the start of the study from the Health Research Ethics Committee of FMCO for the use of archived samples previously obtained under adequate written informed consent and ethics approval scope (Ref: FMC/OW/380/VOL.CXIV/102, 13 April 2021) for future research purposes. The authors did not have access to information that could identify individual participants during or after data collection.

## Results

### Participant and sample characteristics

**Fig 1:**
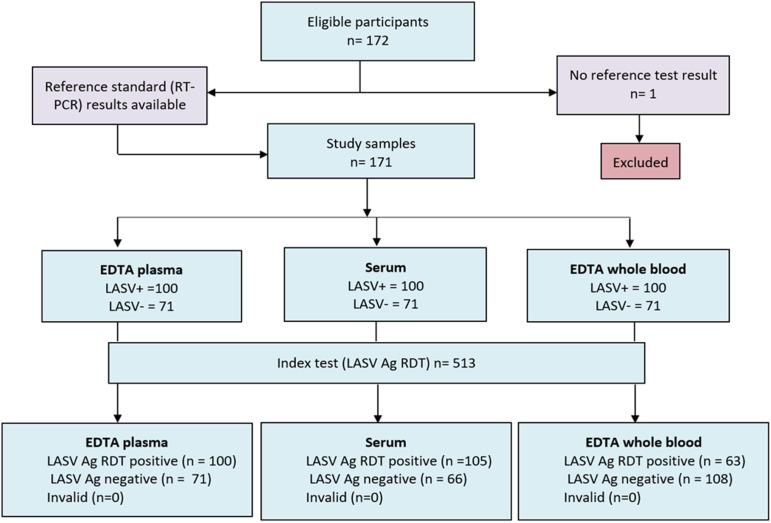
Sample flowchart.

There was a total of 172 eligible participants, yielding 101 LASV-positive and 71 LASV-negative samples (Fig 1). One participant was excluded due to a missing reference result. Participant characteristics are shown in Table 1.

**Table 1:** Participant characteristics.

Reference test result	Category	LASV positive	LASV negative
**n(%)**	100 (58.0)	71 (42.0)
**Sex, n(%)**	Male	53 (53.0)	33 (46.0)
	Female	46 (46.0)	37 (52.0)
**Disease severity, n(%)**	Not ill	2 (2.0)	5 (7.0)
	Mildly ill	73 (73.0)	50 (70.0)
	Gravely ill	22 (22.0)	16 (23.0)
**Age, n(%)**	<18	5 (5.0)	3 (4.0)
	18-29	22 (22.0)	18 (25.0)
	30-39	27 (27.0)	16 (23.0)
	40-49	19 (19.0)	15 (21.0)
	50-59	14 (14.0)	9 (13.0)
	≥60	13 (13.0)	10 (14.0)

### Sensitivity and specificity versus reference test

Analysis of the ReLASV Ag-RT compared to qRT-PCR performed on plasma samples showed a sensitivity of 65.0% (95% CI 55.3-73.6) and a specificity of 50.7% (95% CI 39.3-62.0), with a diagnostic odds ratio of 1.91 (95% CI 1.87-1.95), indicating a relatively weak discriminatory power of the index test (Table 2). The performance of the index test using serum samples was similar to that of plasma (Table 2). Significantly lower sensitivity was observed with whole blood, although the specificity (66.2%) on whole blood was slightly higher, with a diagnostic odds ratio of 50.3 (95% CI 42.9-57.7). Sensitivity trended upward with severity of illness and decreasing Ct values, but all confidence intervals overlapped. There were no statistically significant performance differences by sex.

**Table 2:** Subgroup analysis.

Variable		N	TP	FP	FN	TN	Sensitivity% (95% CI)	Specificity % (95% CI)	Accuracy % (95% CI)
**Sample type**	Plasma	171	65	35	35	36	65.0 (55.3-73.6)	50.7 (39.3-62.0)	59.1 (51.6-66.2)
	Serum	171	68	37	32	34	68.0 (58.3-76.3)	47.9 (36.7-59.3)	59.7 (52.2-66.7)
	Whole blood	171	39	24	61	47	39.0 (30.0-48.8)	66.2 (54.6-76.1)	50.3 (42.9-57.7)
**Disease severity**	Not ill	7	1	3	1	2	50.0 (9.5-90.6)	40.0 (11.8-76.9)	42.9 (15.8-75.0)
	Mildly ill	123	46	23	27	27	62.3 (40.7-77.3)	53.8 (31.0-73.8)	58.8 (42.2-70.3)
	Gravely ill	38	18	9	4	7	81.8 (61.5-92.7)	43.8 (23.1-66.8)	65.8 (49.9-78.8)
**Ct values**	Ct < 35	83	54	0	29	0	65.1 (54.3-74.4)	-	65.1 (54.3-74.4)
	Ct < 30	37	29	0	8	0	78.4 (62.8-88.6)	-	78.4 (62.8-88.6)
	Ct < 25	17	14	0	3	0	82.4 (59.0-93.8)	-	82.4 (59.0-93.8)
**Sex**	Male	86	36	16	17	17	67.9 (54.5-78.9)	51.5 (35.2-67.5)	61.6 (51.1-71.2)
	Female	83	28	19	18	18	60.9 (46.5-73.6)	48.7 (33.5-64.1)	55.4 (44.7-65.6)

CI, confidence interval; FN, false negative; FP, false positive; TN, true negative; TP, true positive.

## Discussion

In this study, we found low clinical sensitivity and specificity of the ReLASV Ag-RT compared to the PCR reference test for LF. There are at least three other published evaluations of this test, with somewhat discrepant results, two of which were also conducted in Nigeria. In Abakaliki, Nigeria, Elsinga *et al* conducted a prospective study using fresh samples and found specificity on testing plasma over 90%, while sensitivity (50%) was similar to that noted in our study, dipping down to 10% when testing capillary blood at the bedside, with a poor correlation with viral load [16,17]. In contrast, in a study conducted during a LF outbreak in central Nigeria (which included investigators from the ReLASV Ag-RT manufacturer), Boisen *et al* reported considerably better performance, with a sensitivity of 83.3% and specificity of 92.8% when compared to composite results of two qPCR assays, using predominantly LASV lineage II samples [8]. All samples with Ct values below 22 on both qPCR assays were positive. The higher pre-test positive predictive value associated with an outbreak may have positively impacted the observed sensitivity in this study [18]. Boisen *et al* also reported high sensitivity (90%) and specificity (100%) of a previous version of the ReLASV Ag-RT based on paired monoclonal antibodies to LASV lineage IV in Sierra Leone, although the diagnostic standard used in the study, combining clinical suspicion and the results of immunoassays and qPCR, was not always clear [19].

Although we did not, other investigators found a specificity of the ReLASV Ag-RDT over 90%, suggesting that it could potentially be used in the confirmation of LF. However, further evaluation should be undertaken, as well as corresponding diagnostic algorithms, if the test is to be used in this manner. Independent evaluation of this test for detection of LASV in rodents is also warranted, since it has recently been used for this purpose in Sierra Leone [20]. This would inform the use of the test to facilitate the zoonotic surveillance of LASV and inform the exclusion of rodents from human spaces as a primary preventive measure in the absence of therapeutics and vaccines [20].

We note the following factors, including limitations to our study, which may have contributed to the finding of low diagnostic accuracy, as well as the discrepancy in results between our findings and those of other investigators. First, we did not have genomic data to identify the specific lineages of LASV in the tested samples. Diagnostic accuracy might be reduced if the lineages were predominantly discordant from the LASV lineages II, III, and IV on which the ReLASV Ag-RT is based [21,22]. Nevertheless, it should be noted that the study conducted by Elsinga *et al* in which poor diagnostic accuracy was found was done in an area where LASV lineage II predominates, and thus the result cannot be attributed to a lineage mismatch with the ReLASV RDT [16].

Secondly, as a prospective design is not easily feasible, and best achieved during an outbreak but with an unpredictable magnitude, we chose a retrospective design using a biobank to enable parallel evaluation of the index test on a large population of well-characterized samples. However, the use of archived samples brings possible variations in storage conditions such as temperature fluctuations, freeze-thaw cycles, or prolonged storage durations that could lead to sample degradation and compromise the accuracy of results [23,24]. Nevertheless, since samples in this study were collected within the last 12 months, the likelihood of sample degradation affecting the results is low. There is also the potential for selection bias when using banked biological samples, since the samples may not be representative or capture the diversity of the broader population, thus limiting the generalizability of the study findings [25].

Neither we nor others evaluating the ReLASV Ag-RT controlled for the timing of the sample acquisition during the clinical course. Nor did we measure or correlate with antibody responses to LASV. It is possible that, at least for some samples, the humoral response to LASV infection resulted in the production of antibodies that bound and blocked the LASV NP antigen, making it less detectable by the ReLASV Ag-RT [21,26,27]. Detection of LASV nucleic acid by qPCR would not necessarily be affected by these antibodies, potentially resulting in discordant results and decreased observed sensitivity. Future evaluation of the ReLASV Ag-RT as well as other RDTs for LF should include testing for IgM and IgG antibodies to assess possible antigen-antibody interactions and their impact on test results, although such studies will be challenged by the limited availability of well-validated assays for antibodies to LF as well.

Lastly, considering that this study was powered to prioritize the assessment of the index test’s diagnostic accuracy, we suggest that future diagnostic evaluation studies, where specific subgroups are known to have different clinical performance, should be powered to prioritize the subgroups.

## Conclusion

The low diagnostic accuracy indicated in our and other independent evaluations of the ReLASV Ag-RDT suggests that this test, at least until further developed, refined, and validated, is not suitable for making critical diagnostic or treatment decisions for LF, at least for lineages that commonly circulate in Nigeria. This is unfortunate, considering that the vast majority of confirmed cases of LF occur in Nigeria, and the ReLASV Ag-RDT is presently one of only two RDTs for LF on the market [28]. Furthermore, although in recent years there have been advances in the understanding of the epidemiology of LF and the pre-clinical research and development of vaccines and therapeutics against this dangerous disease, the absence of an accurate low-cost robust diagnostic tool that can be used at or near the bedside in the widespread area of LF endemicity across West Africa presents a major bottleneck to further progress. We encourage the biotech research and development community to continue to engage and expand to meet this challenge and fill the critical gap of an accurate RDT for LF. Lastly, these findings underscore the importance of thoroughly assessing the performance characteristics of tests, to ensure their reliability and accuracy in real-world applications, especially in healthcare settings where diagnostic accuracy is critical.

## Supporting information

S1 Table STARD Checklist. (PDF)

S1 Data Dataset. (XLSX)
